# A Critical Analysis of Concentration and Competition in the Indian Pharmaceutical Market

**DOI:** 10.1371/journal.pone.0148951

**Published:** 2016-02-19

**Authors:** Aashna Mehta, Habib Hasan Farooqui, Sakthivel Selvaraj

**Affiliations:** Public Health Foundation of India, Delhi, India; UNAIDS, TRINIDAD AND TOBAGO

## Abstract

**Objectives:**

It can be argued that with several players marketing a large number of brands, the pharmaceutical market in India is competitive. However, the pharmaceutical market should not be studied as a single market but, as a sum total of a large number of individual sub-markets. This paper examines the methodological issues with respect to defining the relevant market involved in studying concentration in the pharmaceutical market in India. Further, we have examined whether the Indian pharmaceutical market is competitive.

**Methods:**

Indian pharmaceutical market was studied using PharmaTrac, the sales audit data from AIOCD-AWACS, that organises formulations into 5 levels of therapeutic classification based on the EphMRA system. The Herfindahl-Hirschman Index (HHI) was used as the indicator of market concentration. We calculated HHI for the entire pharmaceutical market studied as a single market as well as at the five different levels of therapeutic classification.

**Results and Discussion:**

Whereas the entire pharmaceutical market taken together as a single market displayed low concentration (HHI = 226.63), it was observed that if each formulation is defined as an individual sub-market, about 69 percent of the total market in terms of market value displayed at least moderate concentration. Market should be defined taking into account the ease of substitutability. Since, patients cannot themselves substitute the formulation prescribed by the doctor with another formulation with the same indication and therapeutic effect, owing to information asymmetry, it is appropriate to study market concentration at the narrower levels of therapeutic classification.

## Background

Medicines worth Rs. 74,895 Crore (or USD 12.62 Billion) were marketed in the retail market in India between February, 2013 and January, 2014. Given the fact that, these retail sales were contributed by 534 firms marketing over 2,500 formulations, it can be argued that the pharmaceutical market in India is highly competitive. However, the argument for the competiveness of pharmaceutical market cannot be taken at face value because pharmaceutical market is not a single market but a sum total of a large number of individual sub-markets. This is because medicines used in the treatment of a particular health condition, cannot be substituted with medicines used in the treatment of another health condition.

In traditional economics literature, market has been defined on the basis of cross-price elasticity of demand, price correlations, partial adjustment approach and so on. More recent literature has differentiated between the concept of relevant market used in competition analysis and the traditional economic definition of markets. “Economic markets identify the range of products and geographic areas for which arbitrage keeps price linked. However, this does not establish whether or not a firm or firms have market power.” [1]

The pharmaceutical industry in India has been witnessing increasing consolidation as a result of the recent spate of mergers and acquisitions [2]. The Competition Commission of India (CCI), which is the Indian counterpart of anti-trust bodies such as the United States Federal Trade Commission (USFTC), Commission of the European Communities (EC) regulates (approve or disapprove) proposed mergers and acquisitions in the country. Defining relevant product markets is useful for such competition analyses and anti-trust bodies across the globe, as in India, are time and again faced with the challenge of appropriately defining the relevant market.

For example, relevant product market is defined by the Indian Competition Act, 2002 (amended in 2007) [3] as “a market comprising all those products or services which are regarded as interchangeable or substitutable by the consumer, by reason of characteristics of the products or services, their prices and intended use.” The Horizontal Merger Guidelines of the USFTC assert that market definition helps specify “the line of commerce and the sections of the country where competition concerns can arise” and “to identify market participants and measure market share and market concentration”. Although market definition may not always be a starting point for competition assessment, yet the evaluation of competitive alternatives available to the consumers is always required at some point of the analysis [4].

The factors that may be analyzed so as to determine the plausible appreciable adverse effect on competition in the relevant market are stated in Section 20, sub-section 4 of the Indian Competition Act, 2002 [3], for example Factor (g.) is the “extent to which substitutes are available or arc likely to be available in the market” and factors (c.) and (d.) are “level of combination in the market” and “degree of countervailing power in the market”. Whereas factor (g.) requires the identification of the relevant market, factors (c.) and (d.) require a study of the prevalence of concentration or conversely competition in the market defined as the relevant market. For this purpose, pre and post-merger market shares and concentration indices that make use of market shares are deployed.

Carlton (2007) [5] concluded that market definition and the use of market shares and change in market shares are crude first steps for an analysis. Nonetheless they are useful in eliminating frivolous antitrust cases and hence can be of enormous value to the society. The market can be defined most precisely quantitatively, but the extensive data required for this is usually not available and therefore, markets are often defined based on qualitative information. The purpose of market definition is the identification of market power. It is implicitly assumed while evaluating a merger case that an increase in market concentration leads to increase in prices.

As such, the narrower the market definition, higher the likelihood that a firm would be observed to have market power. Therefore, firms tend to advocate wider market definitions as compared to those adopted by competition authorities [6]. The challenge is to determine how close a potential substitute must be for inclusion in the market. Significant product differentiation helps better define the market boundaries. However it is difficult to agree upon whether the differentiation is significant enough to create a market boundary especially in the absence of clear standards for defining the relevant product market [7]. Baker (2007) [8] concluded that market should be defined based on the consideration of demand substitution alone and other factors like supply substitution can be accounted for in other steps of competition analysis.

Markets need to be defined carefully for carrying out competition analysis, more so for studying competition in the pharmaceutical market. This is because policy decisions with respect to mergers and acquisitions in the pharmaceutical sector are likely to have implications on access to medicines at affordable prices in India, a country where majority of healthcare expenditure is out of pocket, of which a substantial proportion (66.4 percent [computation based on National Sample Survey -68^th^ round]) is spent on medicines alone. Medicines are an indispensable component of health systems and therefore such decisions directly impact public health in the country.

This paper examines the methodological issues with respect to defining the relevant market involved in studying concentration in the pharmaceutical market in India. Further, we have examined whether the Indian pharmaceutical market is competitive.

## Methods

The structure of the Indian pharmaceutical market was studied using PharmaTrac, the sales audit data from AIOCD-AWACS [AIOCD Pharmasofttech AWACS Pvt. Ltd. is a market research company which is a joint venture between All Indian Origin Chemists and Distributors Ltd. (AIOCD Ltd.) and Trikaal Mediinfotech Pvt. Ltd.]. The dataset provides monthly pack-wise sales (values and volumes) in the Indian market. The data is collected from a sample of 18,000 stockists across 23 different regions of the country and then projected to reflect the overall sales in the private sector in the country. Moving Annual Total (MAT) values for the month of January, 2014 were obtained by summing market values for the period February 2013 to January 2014. The market values are in INR. The values were converted into US Dollars using the average exchange rate for the period 1^st^ February, 2013 to 31^st^ January, 2014. The daily exchange rates were obtained from the Reserve Bank of India’s (RBIs) Reference Rate Archive (weblink: https://rbi.org.in/scripts/ReferenceRateArchive.aspx).

We used AIOCD AWACS’s therapeutic classification as the basis of defining the market for the purpose of this analysis. AIOCD-AWACS dataset PharmaTrac organises formulations into 5 levels of therapeutic classification based on the EphMRA system. The European Pharmaceutical Market Research Association (EphMRA) along with Pharmaceutical Business Intelligence and Research Group (PBIRG), develops and maintains anatomical classification of pharmaceutical products since 1971 [9]. Products are classified on the basis of their indications and uses [10]. Further, as per AIOCD AWACS, ‘the classification had been modified at the narrowest level (which is the formulation level), to help companies focus on specific molecules or combinations in the Indian context.’

Market concentration refers to the extent to which the sales in a market are concentrated in the hands of a few firms in the market. The two widely used indicators of market concentration are the four-firm concentration ratio (CR4) and the Herfindahl-Hirschman Index (HHI) [11]. The CR4 is the combined market share of the top four firms with the highest market share in the relevant market. The Herfindahl-Hirschman Index (HHI) is the sum of the squares of the market shares of all the firm in the relevant market.

CR4=S1+S2+S3+S4

$$HHI=S1^{2}+S2^{2}+\ldots ‥+Sn^{2}$$

Where Si = market share of the i^th^ largest firm in the market in terms of market value.

Unlike the CR4 that only takes into account the top four firms in the market, HHI takes into account all the firms in the market, hence providing the complete picture. Moreover, the use of squares of the market shares can be interpreted as using the market share of each firm as its own weight. As a result, higher weights are allocated to firms that command a higher market share in the relevant market. While on the other hand CR4 does not distinguish between the relative sizes of the top four firms and every firm gets the same weightage. HHI is also used as the preferred indicator for market concentration by several anti-trust bodies across the globe to study the impact of mergers and acquisitions on competition in the relevant markets.

HHI was therefore chosen as the indicator for market concentration for this study. Higher HHI implies higher market power with a few firms that may among other things result in higher prices [11]. The highest value of HHI can go up to 100^2^ or 10,000 which is the case of a perfect monopoly. For the purpose of this analysis, high concentration was defined as HHI greater than 2,500, moderate concentration was defines as HHI greater than or equal to 1,500 but less than 2,500 and low concentration was defined at HHI less than 1,500 [12].

We calculated HHI for the entire pharmaceutical market studied as a single market. Further, we calculated the HHI at different levels of therapeutic classification to assess the changes in the market concentration with the changes in the market definition.

## Results and Discussion

### Market Definition and Market Concentration

We observed that PharmaTrac dataset classifies pharmaceuticals in to five levels of therapeutic classification viz. Therapy, Supergroup, Class, Group, and Subgroup or Formulation based on EphMRA classification- Therapy being the broadest and Subgroup the narrowest level of therapeutic classification. The classification includes 17 Therapies, 19 Supergroups, 98 Classes and 357 Groups into which 2583 Formulations (or Subgroups) are classified. The 17 Therapies include Alimentary Tract and Metabolism, Cardiovascular system, Central Nervous System, Respiratory System and so on.

We further observed that market concentration i.e. HHI was 226.63 when the entire pharmaceutical market was studied together as a single market during Feb 2013 to Jan 2014, highlighting extremely low market concentration (see Table 1). However as discussed earlier, the entire pharmaceutical market comprises several sub-markets and therefore concentration should be studied at the appropriate level.

**Table 1:** Market concentration in the entire pharmaceutical market in India studied as a single market.

Entire pharmaceutical market	
Herfindahl—Hirschman Index (HHI)	226.63
Number of firms in the market	534
Total number of brands	35272
Total market value (Rs. Crore)	74895.03(USD 12.62 Billion)

Note: Market values based on Moving Annual Total (MAT) for the month of January, 2014

Source: Author’s computations based on AIOCD-AWACS market dataset PharmaTrac

We studied market concentration at different levels of therapeutic classification from the broadest level (therapy) to the narrowest level (formulation) (Table 2). We observed that only 1 out of the 17 therapies was observed to have high concentration. This therapy accounted for just 0.23 percent of the total pharmaceutical market in terms of sales value. The remaining therapies accounting for 99.77 percent of the market, demonstrated low concentration. At the Supergroup level, only 2 out of 19 (~11 percent) Supergroups displayed high concentration. These Supergroups together accounted for 1.39 percent of the total pharmaceutical market.

**Table 2:** Market concentration at different levels of market definition.

	Market concentration	Number (percentage)	Cumulative market share (%)
**Therapy Level**	High (HHI≥2500)	1 (6%)	0.23
	Moderate (2500>HHI≥1500)	0	0
	Low (HHI<1500)	16 (94%)	99.77
	Total	17	100.00
**Supergroup Level**	High (HHI≥2500)	2 (11%)	1.39
	Moderate (2500>HHI≥1500)	1 (5%)	1.51
	Low (HHI<1500)	16 (84%)	97.09
	Total	19	100.00
**Class Level**	High (HHI≥2500)	27 (28%)	5.66
	Moderate (2500>HHI≥1500)	15 (15%)	5.18
	Low (HHI<1500)	56 (57%)	89.15
	Total	98	100.00
Group Level	High (HHI≥2500)	173 (48%)	14.09
	Moderate (2500>HHI≥1500)	85 (24%)	14.87
	Low (HHI<1500)	99 (28%)	71.04
	Total	357	100.00
**Formulation Level**	High (HHI≥2500)	2230 (86%)	48.25
	Moderate (2500>HHI≥1500)	225 (9%)	20.70
	Low (HHI<1500)	128 (5%)	31.05
	Total	2583	100.00

Note: Market values based on Moving Annual Total (MAT) for the month of January, 2014

Source: Author’s computations based on AIOCD-AWACS market dataset PharmaTrac

At the class level, we observed that 27 (~28 percent) out of a total of 98 classes had high market concentration. The cumulative market share of these classes was about 6 percent of the total market. Another 15 (~15 percent) classes accounting for another 2.18 percent of the total market in trems of sales value had moderate concentration.

At the group level, 173 (~49 percent) out of the 357 groups demonstrated high market concentration. These groups taken together accounted for 14 percent of the market. Another 85 (~24 percent) groups were observed to have moderate concentration. The cumulative market share of these groups was 15 percent.

Lastly market concentration was studied at the level of individual formulations. 2230 (~86 percent) out of 2583 formulations were observed to have high market concentration. These formulations accounted for 48 percent of the total market in terms of sales value. Another 225 (~9 percent) formulations demonstrated moderate concentration. The cumulative market share of these formulations was 21 percent.

### Cardiac medicines- a case in point

We argue that the treatment of a particular health condition say, cardiovascular ailments cannot be substituted with the medicines used to treat another health condition say, respiratory ailments. Hence, for the competition and concentration analysis the market for Cardiovascular medicines should be treated separately from the market for medicines used to treat other health conditions. We have taken Cardiac medicines as an illustrative example to explain therapeutic classification. The market for Cardiac medicines which is worth Rs. 9248.55 Crores (USD 1.56 Billion), is the second largest therapeutic segment in the Indian pharmaceutical market with the market share of 12.35 percent. At supergroup level this market displayed low concentration with an HHI of 388.9 (Table 3).

**Table 3:** Market concentration at the Supergroup level.

Supergroup	Market Value in Rs. Crore (USD Billion)	No. of companies	HHI
Anti-Infectives	12600.33 (2.12)	402	431.23
**Cardiac**	**9248.55 (1.56)**	**199**	**388.90**
Gastro Intestinal	8436.00 (1.42)	431	273.61
Vitamins / Minerals / Nutrients	6697.86 (1.13)	428	241.24
Respiratory	5880.57 (0.99)	383	599.43
Pain / Analgesics	5407.86 (0.91)	418	271.81
Anti Diabetic	5209.09 (0.88)	124	579.04
Neuro / Cns	4687.71 (0.79)	249	863.11
Gynaecological	4675.91 (0.79)	342	313.04
Derma	4121.56 (0.69)	265	528.10
Ophthal / Otologicals	1353.71 (0.23)	121	848.87
Hormones	1278.70 (0.22)	129	831.67
Vaccines	1132.78 (0.19)	33	1512.57
Anti-Neoplastics	980.00 (0.17)	66	860.32
Others	907.39 (0.15)	422	285.32
Blood Related	898.38 (0.15)	163	402.64
Anti Malarials	617.72 (0.10)	107	2598.05
Sex Stimulants / Rejuvenators	426.80 (0.07)	58	2216.53
Stomatologicals	334.11 (0.06)	100	1420.15

Note: Market values based on Moving Annual Total (MAT) for the month of January, 2014

Source: Author’s computations based on AIOCD-AWACS market dataset PharmaTrac

We have treated the Supergroup level as out starting point and Cardiac as a single Supergroup comprising 12 classes including anti-thrombotic agents. (Table 4). We observed among theses 12 classes, three classes (Other Cardiovascular Products, Cerebral and Peripheral Vasotherapeutics and Anti-hypertensives) had high market concentration. The class Anti-anginals was moderately concentrated and remaining 8 classes displayed low market concentration. We further observed that the HHI for Agents Acting on the Renin-angiotensin System, the class with the highest market value (Rs. 2158.46 crores or USD 0.36 Billion) within the Supergroup cardiac was just 536.12 (Table 5). However, among 11 groups within the class Agents Acting on the Renin-angiotensin System two groups (Renin Inhibitor and Renin Inhibitor, Combined with Anti-hypertensives) had HHI of 10000 i.e. there is perfect monopoly in these markets. Further, three more groups (Ace Inhibitors+Statins/Fibrates, A Inhibitor, Combined with Anti-hypertensives and Angiotensin-II Antagonists+Statins/Fibrates) displayed high market concentration. Three groups viz. Angiotensin-II Antagonists+ACE Inhibitors, ACE Inhibitors, Combined with Diuretics and ACE Inhibitors, Plain demonstrated moderate market concentration. The remaining three groups were observed to be unconcentrated. The market for the group with the second highest market value within the class under study, Angiotensin-II Antagonists-Plain too was observed to be unconcnetrated with HHI 692.47.

**Table 4:** Market concentration in different Classes within the Supergroup Cardiac.

	Supergroup/Class	Market Value in Rs. Crore (USD Billion)	No. of companies	HHI
Suoer group	Cardiac	9248.55 (1.56)	199	388.90
**Class**	**Agents Acting On The Renin-Angiotensin System**	**2158.46 (0.36)**	**97**	**536.12**
	Beta-Blocking Agents	1718.16 (0.29)	121	518.91
	Lipid-Regulating / Anti-Atheroma Preparations	1648.25 (0.28)	102	676.44
	Antithrombotic Agents	1243.99 (0.21)	80	626.21
	Calcium Antagonist	1169.66 (0.20)	93	484.68
	Cardiac Therapy	586.35 (0.10)	68	653.79
	Diuretics	283.71 (0.05)	54	1023.05
	Anti-Hypertensives	189.69 (0.03)	26	2815.27
	Anti-Anginals	173.07 (0.03)	23	2340.16
	Other Cardiacvascular Preparations	59.85 (0.01)	30	983.06
	Cerebral And Peripheral Vasotherapeutics	9.20 (0.002)	8	3416.22
	Other Cardiovascular Products	8.16 (0.001)	7	3420.55

Note: Market values based on Moving Annual Total (MAT) for the month of January, 2014

Source: Author’s computations based on AIOCD-AWACS market dataset PharmaTrac

**Table 5:** Market concentration in different Groups within the Class Agents Acting on the Renin-Angiotensin System.

	Supergroup/Class/Group	Market Value in Rs. Crore (USD Billion)	No. of companies	HHI
Supergroup	Cardiac	9248.55 (1.56)	199	388.90
Class	Agents Acting On The Renin-Angiotensin System	2158.46 (0.36)	97	536.12
Group	Angiotensin Ii Antagonists + Diuretics	774.82 (0.13)	89	651.89
	**Angiotensin-Ii Antagonists, Plain**	**774.04 (0.13)**	**84**	**692.47**
	Ace Inhibitors, Plain	366.16 (0.06)	55	1570.80
	Angiotensin-Ii Antagonists, Combined With Anti-Hypertensives	123.70 (0.02)	24	997.86
	Ace Inhibitors, Combined With Diuretics	90.34 (0.02)	39	1595.04
	Angiotensin Ii Antagonists + Ace Inhibitors	13.04 (0.002)	17	2493.65
	Ace Inhibitors + Statins / Fibrate	6.40 (0.001)	6	8721.34
	Ace Inhibitors, Combined With Anti-Hypertensives	4.22 (0.001)	7	5436.23
	Angiotensin-Ii Antagonists + Statins / Fibrate	4.18 (0.001)	9	2648.29
	Renin Inhibitor	1.10 (0.0002)	1	10000
	Renin Inhibitor, Combined With Anti-Hypertensives	0.45 (0.0001)	1	10000

Note: Market values based on Moving Annual Total (MAT) for the month of January, 2014

Source: Author’s computations based on AIOCD-AWACS market dataset PharmaTrac

Finally each of the six formulations within the group Angiotensin-II Antagonists-Plain was studied as an individual market. The HHIs ranged from only 789.72 for Olmesartan to as high as 5502.93 for Candesartan. Out of the six formulations being marketed within the group under study, two formulation viz. Olmesartan and Telmisartan displayed low concentration, one formulation i.e. Losartan displayed moderate concentration and three formulations viz. Irbesartan, Candesartan and Valsartan displayed high concentration (Table 6).

**Table 6:** Market concentration in different Formulations within the Group Angiotensin-II Antagonists, Plain.

Therapeutic classification	Market Value in Rs. Crore (USD Millions)	No. of companies	HHI
Supergroup			
Cardiac	9248.55(1558.87)	199	388.90
Class			
Agents Acting On The Renin-Angiotensin System	2158.46(363.82)	97	536.12
Group			
Angiotensin-Ii Antagonists, Plain	774.04(130.47)	84	692.47
Formulations			
TELMISARTAN | C9D4	388.20(65.43)	65	1309.76
LOSARTAN | C9D3	184.35(31.07)	49	1651.16
OLMESARTAN | C9D7	175.71(29.62)	30	789.72
VALSARTAN | C9D5	20.61(3.47)	4	3427.12
IRBESARTAN | C9D2	5.00(0.84)	3	6214.52
CANDESARTAN | C9D1	0.17(0.03)	4	5502.93

Note: Market values based on Moving Annual Total (MAT) for the month of January, 2014

Source: Author’s computations based on AIOCD-AWACS market dataset PharmaTrac

## Discussion

We observed significant changes in market concentration in the Indian pharmaceutical market as the market definition was altered from broader ATC levels to narrower ATC levels. Whereas the entire pharmaceutical market taken together as a single market displayed low concentration, it was observed that if each formulation is defined as an individual sub-market, the markets for 86 percent of the total formulations having a cumulative market value of over Rs. 36000 crores demonstrated high market concentration and those of another 9 percent of the formulations having a cumulative market value of over Rs 15500 crores demonstrated moderate market concentration. In other words, together about 95 percent of the total market in terms of the number of formulations and about 69 percent of the total market in terms of market value displayed at least moderate concentration for the period under study.

The market for cardiac medicines was used as an illustration to explain the changes in market concentration with the changes in market definition. As Table 6 demonstrates, the market for all Cardiac medicines taken as a single market (Supergroup level), was observed to have low concentration with HHI 388.90. The Class, Agents Acting on the Renin-Angiotensin System had an HHI of 536.12. Within this class, the Group, Angiotensin-II Antagonists, Plain had an HHI of 692.47. Finally, at the formulation level within this group, we observed that the HHI ranged from 789.72 for Olmesartan to 5502.93 for Candesartan. It is clear that the value of HHI increased at each stage as we systematically progressed towards narrower levels of ATC classification. It is obvious from the analysis, that an incorrect market definition can be expected to lead to erroneous conclusions about the state of competition in the pharmaceutical market. Defining the relevant market appropriately, is key to understanding competition and concentration in pharmaceutical market.

As noted earlier, ATC classification forms the basis of competition analysis in the pharmaceutical market as medicines intended for different indications cannot be used interchangeably. Product differentiation based on intended use and therapeutic value, helps ascertain market boundaries. These market boundaries are clearer at broader levels of therapeutic classification (therapy, supergroup level). However, as we move towards narrower levels of therapeutic classification (class, group, formulation level), these boundaries start to blur.

The choice of correct market definition is tricky for pharmaceuticals because unlike several other markets, the pharmaceutical market is not governed by the idea of consumer sovereignty. The prescriber makes the choice of the medicine to be consumed by the patient, the end user. In the absence of specialised information, the patient or even the pharmacist is usually unable to substitute the medicine prescribed by the doctors in view of the health condition, with another formulation, even with the exact same indication or the same mechanism of action without first consulting with the prescribing doctor, owing to acute information asymmetry.

It is recognized that the spectrum of disease and their symptoms are unique for each patient. Disease management too is personalized to suit the individuals’ requirements through the selection of specific formulations, their dosage form and strengths so as to achieve maximum therapeutic benefit. In addition, the pharmacodynamic response of a patient’s body to a medicine varies due to genetic variability, co-morbidities and co-medication. Hence, the treating physician chooses the appropriate medicine in the suitable dosage form and strength, taking into account all the possible variability depending among other things on patient history.[13] Owing to these factors, the likelihood of substitution between formulations by patients is negligible. This argument is further strengthened by the fact that the standard treatment guidelines and consensus statements released by professional bodies for specific diseases explicitly mentions treatment of choice (i.e. formulations, which are preferred over other formulations within the same group and are theoretically substitutable). For example, the treatment of choice for hypertension for young patients, pregnant women and elderly differs significantly. [13]

In addition, price sensitivity of the consumer is often used as a yardstick for defining the market. It must be noted that the choice of medicine by the doctor is often not made based on the consideration of prices of different medicines. Since the doctor, chooses the medicine but does not pay for it [14]. Therefore the demand for medicines is more often than not price insensitive.

Finally, it may be noted that no straightforward generalisations can be drawn with regard to the decision concerning market definition. Hence, depending on the research question at hand, the choice of market definition may vary. For instance, in the case of medicines intended for specialised health conditions such as cancer, a narrower market definition should be preferred as the patients have absolutely no consumer sovereignty in such markets and the treatment protocols are personalized with regard to stage of disease. On the other hand, a broader market definition could be used for studying over-the-counter (OTC) medicines, as patients enjoy relatively higher consumer sovereignty in the market for OTC products compared to prescription medicines.

In fact anti-trust bodies take a case by case approach for defining the market for competition analysis depending on the country specific market characteristics and the therapeutic segment under study, among other things. For instance, the Commission of the European Communities (EC), in the Novarts/Hexal case (COMP/M.3751) noted that “*the 3*^*rd*^ *ATC level allows medicines to be grouped in terms of their therapeutic indications, i.e. their intended use. This level is generally used as the starting point for defining and enquiring about market definition in competition cases. However, it is appropriate to carry out analyses at other ATC levels, or a mixture thereof, if the circumstances of a case show that sufficiently strong competitive constraints faced by the undertakings involved are situated at another level, and that, therefore, there are indications that the third ATC level does not lead to a correct market definition*.” [15]

The EC pointed out in the *Teva/Barr case (COMP/M*.*5295) that “…market investigation has indicated that—in particular for drugs purchased by hospitals—competition primarily takes place between drugs based on the same molecule*. *A majority of hospitals queried by the Commission stated that*, *in particular for serious illnesses*, *they would not consider switching from drugs based on one molecule to drugs based on another*, *even if the price for the molecule would increase significantly*.*”*[16]

The Competition Commission of India (CCI) in its recent evaluation of the merger of Sun Pharmaceutical Industries Limited and Ranbaxy Laboratories Limited (C-2014/05/170) noted that “*the pharmaceutical drugs within a group may not be substitutable because of differences in the intended use*, *mechanism of action of the underlying molecule*, *mode of administration*, *contra-indications*, *side effects etc*.*”* Further, keeping in mind the dynamics of the Indian pharmaceutical market, the Commission emphasized that “…*in generics markets*, *competition primarily takes place between different brands based on the same molecule*.*”* [17]

Therefore, it may be argued in the context of the Indian pharmaceutical market, for the above case study of cardiac medicines, the different companies marketing several brands of the same formulation compete with each other and companies marketing different formulations even within the same group or class do not necessarily compete with each other.

## Conclusion

The results demonstrate that the broader the market definition, the lower the market concentration and as the definition of the market is narrowed down, market concentration increases. Therefore the relevant market should be carefully defined so as to draw meaningful inferences with regard to market concentration.

As a rule of thumb, market should be defined taking into account the ease of substitutability among medicines based on the understanding of how the forces of competition operate in the market under study. In other words, market should be defined based on the practical substitutability between formulations and not just theoretical substitutability. Patients cannot, on the basis of their own judgement substitute the formulation prescribed by the doctor with another formulation that may be theoretically substitutable with the prescribed formulation owing to similar indication and therapeutic effect, as a result of information asymmetry. In the best case scenario, patients may be able to choose from among the different brands of the same formulation. We observed that the pharmaceutical market, when defined by treating each individual formulation as a separate market, is far from competitive. It is in fact significantly concentrated.
